# Concerns or Desires Post-Pandemic: An Extended MGB Model for Understanding South Korean Residents’ Perceptions and Intentions to Travel to China

**DOI:** 10.3390/ijerph18052542

**Published:** 2021-03-04

**Authors:** Guanghui Qiao, Xiao-li Zhao, Luqi Xin, Seokchool Kim

**Affiliations:** 1 School of Tourism and Urban-Rural Planning, Zheshang Research Institute, Zhejiang Gongshang University, Hangzhou 310018, China; qgh@zjgsu.edu.cn; 2 Tourism Management Discipline, Paichai University, Daejeon 35345, Korea; xiaoli84@daum.net; 3 Department of Tourism Festival and Resort Management, Paichai University, Daejeon 35345, Korea; sckim@pcu.ac.kr

**Keywords:** COVID-19, concern, desire, mass media, extended MGB, travel intention, South Korea, China

## Abstract

In this study, we examined South Korean residents’ travel-related behavioural intention for mainland China post-COVID-19 using an extended model of goal-directed behaviour. To do so, we integrated South Korean residents’ perceptions of country image (PCI), mass media, and concerns about travel into the framework of the original model of goal-directed behaviour (MGB). Structural equation modelling was used to identify the structural relationships among the latent variables. The results show that mass media had a positive influence on South Korean residents’ perception of China’s image, a negative influence on residents’ concerns, and a positive influence on residents’ behavioural intentions for travel overseas. Meanwhile, PCI had a positive influence on residents’ attitude towards travel overseas. The theoretical and practical implications of the study are discussed.

## 1. Introduction

**Figure 1:**
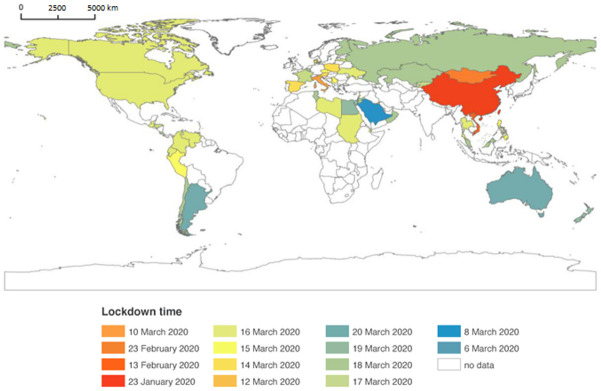
Lockdown policies worldwide by date and country (resources shown in Supplementary Materials).

The COVID-19 pandemic has severely disrupted the global economy including the tourism industry. Proclaimed as a pandemic on 11 March 2020 by the World Health Organization (WHO) [1], COVID-19 has evolved into the largest public health crisis since the Spanish Flu pandemic of 1918/19 (Table 1). As of February 2021, the number of people infected continued to grow. To combat the pandemic, numerous countries implemented lockdown strategies with varying degrees of success (Figure 1). Strategies adopted as part of lockdown policies have included the suspension of international flights, shutting of restaurants, museums, sporting events, religious services and tourist attractions. Tourism, an industry that has relatively low levels of resilience to long-running crisis events has been severely affected by the pandemic. The severe acute respiratory syndrome (SARS) crisis of 2002–2003 provides a useful example of how a large public health crisis can affect the tourism industry. SARS caused direct economic losses to the tourism industry in China, Vietnam, Hong Kong and Singapore. Lost GDP in the region exceeded $20 billion, and 2.8 million tourism sector employees lost their jobs [2]. The impact of the COVID-19 pandemic on the global economy and tourism has far exceeded the impact of SARS.

Wuhan, the first city in China to be affected by COVID-19 was able to eliminate severe acute respiratory syndrome coronavirus 2 (SARS-CoV-2) through stringent non- pharmaceutical interventions (NPI) including a strict lockdown. To prevent the spread of SARS-CoV-2 to the remainder of China, the national government imposed strict government control of all movement into and out of Hubei province where Wuhan is located. The government also provided significant support for citizens in the province. By late March 2020, the elimination strategy had succeeded and the national government began to encourage citizens to participate in tourism and leisure activities as a way of promoting e economic recovery. Many local governments also introduced measures and policies to stimulate tourism consumption. For example, from April 2020, all A-level scenic spots in Ningxia were reopened and visits were free for medical staff, current veterans, and police officers from all over the country. Zhejiang introduced a scheme to provide cultural and tourism vouchers with a total value of 1 billion yuan and as an additional measure provided a large “red envelope” of 100 million yuan for cultural tourism consumption. Beijing announced a tourism development subsidy fund of 345 million yuan that was made available to tourism companies to respond to the pandemic. Firms were also encouraged to seek subsidies from the National Tourism Development Fund of the Ministry of Culture and Tourism. These measures have contributed to the recovery of China’s domestic tourism market. For example, during the 7-day Golden Week national holiday that commenced on 1 October 2020, China’s domestic tourism destinations received 115 million visitors and generated 47.56 billion yuan in tourism revenue [3]. While China’s domestic tourism gradually recovered through late 2020 and early 2021, the tourism sector in many other countries affected by COVID-19 continued to suffer low demand. As of early 2021, international tourism exchanges remain largely frozen with this sector of the tourism industry suffering a higher level of impact than domestic tourism. 

A small number of countries including Greece [4] have attempted to revive international tourism but with limited success. Large scale resumption of international tourism flows is unlikely to resume until high levels of vaccination are achieved in generating regions and destinations. Even with widespread vaccinations, the willingness of people to resume international travel will require a high degree of confidence that target destinations are safe. The ability of people to make their own assessment of the level of safety available in target destinations will depend on their access to and trust in the media. 

**Figure 2:**
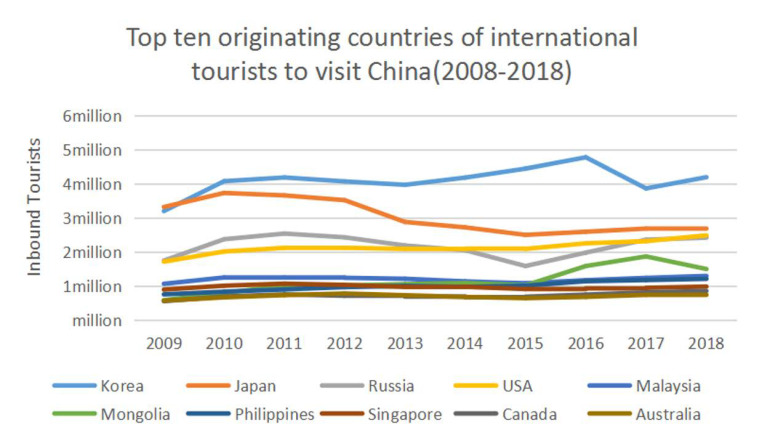
Top 10 originating countries of international tourists visiting China (2008-2018; Source: National Bureau of Statistics (2020) https://data.stats.gov.cn/ (accessed on 20 November 2020).

Bilateral tourism between China and South Korea commenced during the 1988 Seoul Olympic Games when the ban on travel from South Korea to China was lifted. From the first official contact in 1992 up to 2019, mutual visits between Chinese and South Korean tourists grew rapidly and the two countries have become important bilateral tourism destinations (Figure 2). South Korea has been one of China’s main source of inbound tourists. The resumption of post-COVID-19 travel is likely to depend on the level of trust that tourists from both countries in the bilateral pairing have in the health systems of their intended destination. 

Human society had experienced many public health crises. However, given the influence range, the death toll, the duration time and the profound impact on people’s work and life, the COVID-19 pandemic is the most violent crisis. COVID-19 pandemic also led to an unprecedented loss of tourism and hospitality industry.

The aim of this study aim is to investigate the willingness, attitudes, concerns, and behavioural intentions of South Koreans to visit China post-COVID-19. We asked South Korean respondents to evaluate China’s national image post-COVID-19. We also attempt to predict the development trend of international tourism within the context of the COVID-19 pandemic.

Based on these research aims, we developed the following research questions:

1. What is the perception of the national image of China following the coverage of COVID-19 by the mass media in Korea?
2. What are the desires concerning travel to China post-COVID-19?
3. What are the concerns about travelling to China post-COVID-19?
4. What is the behavioural intention to travel to China post-COVID-19?
5. What are the preferred types of overseas destinations post-COVID-19?

To better understand these questions, we developed an extended model of goal-directed behaviour (EMGB). Specifically, we inserted mass media effect (MM), perception of country image (PCI), and concerns into the original MGB (attitude, subjective norm, perceived behavioural control (PBC), positive emotion, negative emotion, desires, past experience and behavioural intention) in the context of the COVID-19 pandemic. Very few studies have investigated potential international tourists’ behavioural intention following a crisis event such as COVID-19. We asked a sample of South Korean residents to explain their decision-making process by applying the EMGB. We hope that the results of this study will provide insights into and an understanding of Korean residents’ thinking and intended outbound tourism decision-making processes within the context of COVID-19.

## 2. Literature Review

### 2.1. The Pandemic and Tourism

The rapid shift towards globalization in recent decades has resulted in increasingly close political, economic, cultural, and social ties between countries. This has been reflected in the reduction of trade barriers and the creation of global supply chains. Another result of globalisation has been a swift increase in populations flows between nations particularly in the areas of business and tourism.

Tourism is an industry concerned with the movement of people both domestically and internationally. While the surge in demand for travel has accelerated the scale of tourism flows [5], the tourism industry is highly sensitive to external crisis events such as pandemics, terrorist activities, natural disasters financial crises, wars and other factors that affect international tourism flows. Public health concerns can generate considerable fluctuations in demand for international travel [6]. In situations such as those that have occurred during the COVID-19 pandemic, virus transmission routes and speed of infection, the sophistication and complexity of transportation networks, the characteristics of population movement, urbanisation trends, the quality of national medical and health services, and pandemic control measures can affect how both the supply and demand sides of tourism are affected [7].

During the 21st century, humanity has experienced five infectious disease outbreaks: SARS in 2002–2003, H1N1 in 2009, MERS-COV in 2012, Ebola in 2000–ongoing, and COVID-19 commencing in 2020. H1N1 and COVID-19 may be classified as pandemics, while MERS, SARS, and Ebola were regional epidemics. SARS was first discovered in Guangdong Province, China in November 2002, from where it gradually spread to other parts of the world in 2003 via travel. The WHO and the worldwide media issued travel warnings for areas that were hardest hit by SARS. Due to SARS, nearly three million people in the tourism industry lost their jobs, the flow of tourists to Asia decreased by 70% [8], and the number of international tourists arrivals declined for the first time in the 21st century [9]. The global spread of H1N1 in 2009 was first detected in Southern California. H1N1 caused approximately 284,000 deaths and led to the loss of nearly 1 million inbound tourists to Mexico during the five months that it lasted [10]. As a result of SARS in 2003 and H1N1 in 2009, inbound travel to China declined by 22.7% for all tourists and 10.3% for South Korean tourists [11]. This was a significant loss because South Korea had been the largest source of tourists for China for the previous 10 years. Of the pre-COVID-19 regional epidemics and pandemics, only SARS led to a reduction in the overall number of international arrivals [12]. COVID-19 has led to a much larger and long-running fall in global tourism flows.

COVID-19 is not as highly contagious as some disease such as measles; nor does it have the 50–80% mortality rate of Ebola. The most significant problem with SARS-CoV-2 is that symptoms may not appear for some days after infection [13] and in some cases a person may show no symptoms. Because infected individuals may be either presymptomatic or asymptomatic, they may not see the need to self-quarantine or take other protective measures and thus unknowingly infect others [14]. Additionally, because prevention and control measures vary between countries, COVID-19 spread rapidly. On 9 January 2021, there were 86,749,940 confirmed cases of COVID-19 and 1,890,342 deaths worldwide [15]. Since March 2020, international travel bans and restrictions on public activities have been in effect for 90% of the world’s population with the result that the global tourism industry has stagnated [9]. 

As of January 2021, COVID-19 had been effectively controlled in China and domestic tourism has begun to recover. However, due to the impact of pandemic control measures, tourists’ concerns for their safety and other potential tourism risks, the market potential for inbound tourism was not favourable. Given this context, we will attempt to explain and predict possible future trends in South Korean travel to China. The results provide a useful reference point for policies designed to encourage the resumption of post-COVID-19 international tourism flows.

### 2.2. The Relationship between Mass Media’s Effect, Perceptions of Country Image, Desire, Concerns, and Behavioural Intention

#### 2.2.1. Mass Media’s Effect

There are two interpretations of the meaning of mass media. One is the view that mass media are the carriers of information transmitted to citizens via books, television, movies, radio, audio and video products, media, magazines, newspapers, periodicals, and the Internet [16]. Another view is that the mass media are entities such as newspapers, websites, major television stations, and other media organizations [16] that are engaged in the collection, processing, and dissemination of information. The main function of the mass media is to handle centralised output, centralised mass production, and one-way massive distribution of information to a large number of controlled audiences [17], to monitor, coordinate, and to entertain [16].

Our daily lives are full of diverse media experiences. Common mass media carriers such as television, radio, magazines, newspapers, websites, and advertisements are an indispensable part of people’s daily lives [18]. Mass media influences the publics’ awareness through disseminating timely, diverse, and large-scale information [19]. Mass media can also confer status and confirm legitimacy. Under special circumstances, mass media can be employed as a means to persuade and mobilise public opinion. In addition to helping to form and maintain public opinion, it is also be used as a tool to provide spiritual rewards and satisfaction [20]. The influence of mass media exists and is not limited to international communication [21], ideology shaping [19], public opinion guidance [19], and information popularisation [22].

Mass media can also influence society and culture [20]. Compared with social media, mass media has greater visibility and influence, and the public has a higher degree of trust in it. Therefore, mass media has a higher degree of influence on public awareness and emotion than social media [23]. According to Barwick et al. [24], information plays an important role in people’s decision-making, an assertion that is further supported by Enikolopov et al. [25], who stated that mass media are the main information source for most people and play an important role in shaping people’s lifestyles and value systems.

#### 2.2.2. Country Image and Perception

National image is a widely studied issue in the fields of communication, political science, and public relations. Scholars divide the national image into two dimensions—macro-national image and micro-national image—and believe that the two are internally related [26,27]. Pappu et al. [28] believe that national image is a construct that includes the national level (macro) and product level (micro). The macro image of a country refers to the sum of descriptive, inferential, and informative beliefs that consumers hold for a country [29]. Micro country image refers to the reputation and impression that consumers have about a country’s products [30]. For example, Pappu et al. [28] measure the macroscopic national image from the three dimensions of economy, politics, and technology, and the microscopic national image from the three dimensions of innovation, prestige, and design.

According to previous research, we can see the differences between the tourism destination image and the national image. The national image refers to peoples’ evaluation of various aspects of a country while the tourism destination image refers to tourists’ perception and evaluation of a specific region, city or country as a tourist destination [31]. In the formation of these two image perceptions, the perception of a country’s image is affected by information on historical events, industrialisation levels, and political and economic systems [32]. The perception of the destination image is affected by the impact of tourism-related information such as tourist attractions, tourist products, and tourist facilities [33]. In the context of tourism, the classification of a national image can also be classified from both macroscopic and microscopic aspects, but the content differs [26,27]. The macro country image includes the national system, national capabilities, national characteristics, environmental conditions, and national relations; while the micro country image includes the tourism environment, attractions, service facilities, and prices and values [26,27].

National image perception is the process of transforming external information into an internal thinking world [34]. Residents’ perception of a country’s image is affected by macroscopic factors such as national strength and culture, as well as by microscopic factors such as friendly personal contact environment and friendly people [35]. An individual’s perception of a country’s image includes three components: cognition, emotion, and intention. The cognitive component is the person’s beliefs about a specific country; the emotional component is the person’s emotional response to a specific country; the intention component is the person’s behavioural intention of purchasing a specific country’s product [26,27]. How then do residents perception of the national image of overseas countries form or take shape? Numerous studies have shown that the local mainstream media play a decisive role in the coverage of target countries [36,37,38]. The relationship between the image of a country and the media can be summarised thus: ‘the image of a country is the image formed by a country in the flow of international news, or the image of a country in the news reports of news media in other countries’ [39]. Only when a country presents a good national image in the international community can it lay a good foundation for the development of politics, economy, culture, and tourism, and be recognised and affirmed by our globalised world. Shaping the image of a country is inseparable from the mass media [40,41]. 

There are two ways to form the image of the country: the others-shaping method and the self-imposing method. A country’s government and various strata shape the countries image through specific behaviours that can be defined as self-shaping. According to Liu [36], ‘the so-called others-shaping’ is an external evaluation and recognition, and a construction derived from the feelings and will of others’. Arana [42] believes that mass media reports construct a virtual reality environment for the audience, which becomes the reference and base that people use to understand reality and make precise judgements. However, this reference and base have obvious limitations. Because they are not completely equivalent to the real world, their degree of homogeneity or contrast will seriously affect the audience’s perception. For the audience, most of whom reside in a large, complex, and rapidly changing society, mass media is a convenient tool and a way to understand the world.

In the case of China, its country’s image is created by the international media and the South Korean media through news reports and speeches [43]. Therefore, based on the literature review, the first research hypothesis is as follows:

**Hypothesis 1** **(H1).** *Mass media’s effect has a positive influence on local residents’ perception of China’s image.*

#### 2.2.3. Mass Media’s Effect, Concerns, and Behavioural Intention

Mass media, a soft power, has created a culture that has changed people’s thinking and behaviour [44]. Meeus et al. [45] explained that prosocial content in the media has a positive influence on the audience. In some countries, the mass media drives crisis management concerning crime, disease, health-related issues, physical equipment failures, weather, cultural barriers, and political crises [46]. A public health emergency is a crisis issue that mass media report on and can influence the public’s concerns about health risks. The high level of consumer use of the mass media makes it possible for a large amount of ambiguous and exaggerated information and rumours to be transmitted to the public, and in doing so, increases their anxieties about becoming infected [47]. Some researchers believe that the information disseminated by the mass media during public health emergencies can cause widespread collective panic. This possibility has been explored by researchers but rarely confirmed [48].

Social media has played an increasingly important role in tourists’ behavioural decisions in recent years [49]. For example, 64% of Americans use social media channels to look for lower-cost leisure, amusement, accommodation, and dining options before beginning their travel. However, because the information on social media is difficult to verify, the mass media’s credibility level with tourists is higher than social media’s concerning their travel intentions and decisions [50]. Therefore, the information provided by the mass media, including its biases, will affect the public’s behaviours concerning preventive measures in a public health emergency. Mass media is also the key influencing factor in the public’s decision to travel and other travel behaviour [51,52,53]. Thus, the second, third, and fourth research hypotheses are as follows:

**Hypothesis 2** **(H2).** *Mass media’s effect has a negative influence on the concerns of local residents for travelling overseas.*

**Hypothesis 3** **(H3).** *Mass media’s effect has a positive influence on the behavioural intention of local residents for travelling overseas.*

**Hypothesis 4** **(H4).** *Concerns have a negative influence on the behavioural intention of local residents for travelling overseas.*

### 2.3. Relationship between Perceptions of Country Image and Attitude

Previous literature has highlighted the complex nature of the image construct, including both cognitive and affective images [54,55,56,57]. Cognitive image refers to the beliefs and opinions that an individual may have regarding a place, whereas affective image refers to emotions and feelings [54,56]. Furthermore, cognitive and affective constructs carry different weights concerning the overall influences of image. Qu et al. [58] found that the cognitive component has a greater influence on preferences for developed and well-known tourism destinations. Baloglu and McCleary [54] found that the affective component has a greater influence on a preference for places that have not been previously visited or have a negative image. The explanation for these findings could be that an individual’s attitude to a place is formed by their perception of an image of a place [59]. Psychologically, attitude is seen as an evaluation of a specific matter with a certain degree of approval or disapproval that represent feelings and thoughts [60]. The construction of attitudes is often reciprocally related to feelings and beliefs, which then serve as a basis for attitudes. Therefore, based on the above literature review, the fifth research hypothesis is as follows:

**Hypothesis 5** **(H5).** *Perceptions of a country’s image have a positive influence on residents’ attitude.*

### 2.4. Model of Goal-Directed Behaviour (MGB) and the Extended Model of Goal-Directed Behaviour (EMGB)

As per the research questions and hypotheses, we explored South Korean residents’ perceptions of China’s national image and their willingness to travel to China in the future. The MGB has been used to explore different tourists’ intention under different tourism contexts. For example, Meng and Choi [61] extended the MGB to uncover the role of authenticity in the formation of tourists’ slow tourism intention. Juschten et al. [62] adopted the MGB to understand metropolitan residents’ intentions to visit nearby alpine destinations in the summer. However, research investigating the relationship between a public health crisis and residents’ intended behaviour is very limited.

#### The History and Development of the Extended Model of Goal-Directed Behaviour

The EMGB is an extended model of goal-directed behaviour based on the theory of planned behaviour (TPB) and the theory of reasoned action (TRA). The TRA posits that subjective norm and behavioural attitude (i.e., attitude towards the behaviour) are the driving factors that affect behavioural intention [63]. To improve the TRA’s predictive ability, Ajzen [64] introduced PBC and formally proposed the TPB. The TPB is used to predict and explain how behavioural attitude, subjective norm, and perceived behavioural control affect individual behavioural intentions and determine the actual behaviour process. However, an individual’s behavioural intention cannot always be explained by the TPB and its three variables. Fortunately, the explanatory power of the TPB can be increased by the addition of more variables. Perugini and Bagozzi (2001) did so by adding three new variables—anticipated emotional factors, past behaviours, and desires—to construct an extended theoretical framework: the MGB. This model includes variables such as attitude, subjective norm, perceived behavioural control, anticipated emotion, desires, frequency of past behaviour, and intentions [65]. The MGB differs from the TPB and TRA models. First, anticipated emotions can be the necessary variables for some specific decision-making behaviours. Second, desire should be influenced by the variables attitude, subjective norm, and perceived behavioural control. Therefore, desire should be a direct motivating factor that affects intended behaviour [66]. Third, previous studies on past behaviour found that it significantly affects tourists’ desire and behavioural intention [65].

With the development of research on tourists’ behaviours, MGB has been applied to a variety of tourism and leisure behaviour [67,68]. For a better understanding of tourists’ behaviour and decision-making process, researchers have again modified the MGB by integrating some new constructs [67,69]. For example, Wang et al. [70] added government policy and protection motivation for preventing smog into the MGB to better discover the formation process of tourists’ behavioural intention for domestic tourism. Meng and Choi [61] integrated the perception of authenticity, knowledge, and information search behaviour into the MGB to provide an insightful understanding of the slow tourist decision-making process. As a result, attitude, subjective norm, positive and negative anticipated emotion, and perceived behavioural control were tested as factors affecting desires and intentions. The study found that the frequency of past behaviour directly and significantly influences desires and intentions [61]. Thus, based on the aforementioned studies, the following hypotheses are proposed:

**Hypothesis 6** **(H6).** *Attitude has a positive influence on desire.*

**Hypothesis 7** **(H7).** *Subjective norm has a positive influence on desire.*

**Hypothesis 8** **(H8).** *Positive anticipated emotion has a positive influence on desire.*

**Hypothesis 9** **(H9).** *Negative anticipated emotion has a negative influence on desire.*

**Hypothesis 10** **(H10).** *Perceived behavioural control has a positive influence on desire.*

**Hypothesis 11** **(H11).** *Frequency of past behaviour has a positive influence on desire.*

**Hypothesis 12** **(H12).** *Desire has a positive influence on behavioural intention.*

**Hypothesis 13** **(H13).** *Frequency of past behaviour has a positive influence on behavioural intention.*

**Figure 3:**
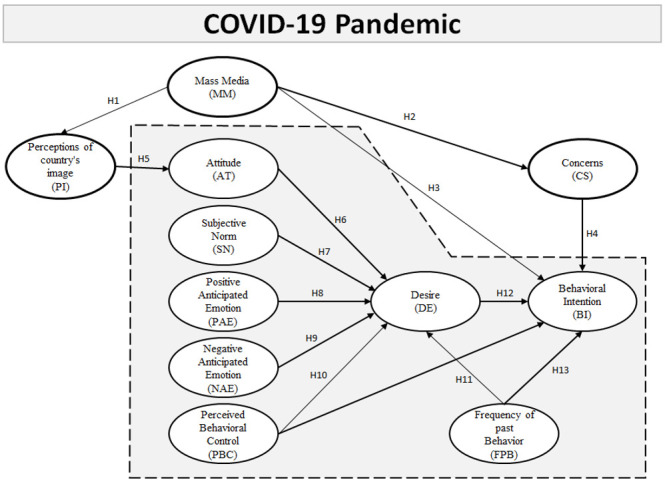
The extended model of goal-directed behaviour (EMGB). Note: The shaded area shows the basic components of the model of goal-directed behaviour (MGB); the three bold ovals are the additional constructs perception of country image (PCI), mass media (MM), and CS that contribute to developing the EMGB. (Note: H1–H13 are hypothesis of this study).

The research model and all hypothesis are shown in the Figure 3 as below.

## 3. Methodology

In this study, we employed a research design using a cross-sectional sample survey aimed at describing the travel intention process of South Korean residents during the COVID-19 pandemic. The survey questionnaire consisted of 53 items:

- Four items on attitude (e.g., ‘Travelling to China is not a positive thing’).
- Three items on the subjective norm (e.g., ‘My family does not support my travel to China’).
- Three items on PBC (e.g., ‘My budget is not enough to support my travel to China’).
- Three items on positive anticipated emotion (e.g., ‘Travelling to China will make me glad’).
- Three items on negative anticipated emotion (e.g., ‘If I could not travel to China, I would feel disappointed’).
- Two items on desire (e.g., ‘I look forward to travelling to China soon’).
- Three items on behavioural intention (e.g., ‘I am planning to travel to China soon’).
- One item on frequency of past behaviour (e.g., ‘If you have travelled in China so far, you have ( ) times).
- Four items on mass media effect (e.g., ‘The media reported the effective controls of the Chinese government in the prevention and treatment of COVID-19’).
- Eight items on concerns (e.g., ‘COVID-19 has made me concerned about handshakes and hugs.)
- Nineteen items on perceptions of country image with the items clustered under five aspects (state institution, national power, national characteristics, environmental management, and international relationship).

Respondents’ perception, travel attitude, and intentions during the COVID-19 pandemic were also measured. All survey items were measured using a 5-point Likert scale (1 = ‘Strongly disagree’ to 5 = ‘Strongly agree’). Excerpts from the survey questionnaire are shown in Table 2.

The original instrument was written in Chinese and translated into Korean. Back translation was recommended when the original questionnaire had to be translated into other languages [71]. In back translation, the questionnaire was translated from the original language (Chinese in this study) into the target language by a bilingual speaker whose native language is the language into which the questionnaire is being translated (Korean in this study). This version was then translated back into the original language by a bilingual translator whose native language is the original language. Translation errors could then be identified when the translated version of the questionnaire was compared with the original version. A committee made up of translators fluent in the two languages administered and discussed the survey questionnaire with regard to translation errors and make modifications until a consensus was reached. This process was followed so that respondents whose native language is not the original language of the questionnaire could comprehend the questions and statements in the translated (Chinese to Korean) questionnaire

This study’s target population were residents of South Korea. Due to COVID-19 related restrictions, the survey was conducted online. The questionnaires were distributed via Facebook, Kakaotalk, and Instagram. These social media platforms are widely used by South Koreans [72]. A pilot online survey was conducted as a development test for the questionnaire. Sixty questionnaires were randomly distributed via Facebook and Kakaotalk, and 50 were returned. The results confirmed that the survey questionnaire was valid.

The online survey was conducted from 10 to 30 September 2020. A total of 1000 questionnaires were distributed online for voluntary respondents and 412 responses were returned, representing a response rate of 41.2%. After eliminating unsuitable answers (e.g., fast responses, and pattern answers), 314 responses were accepted for analysis. The demographic data of the subjects are outlined below and reported in full in Table 3.

- Age—33.8% were male, 66.2% were female; 3.5% were under 18 years old; 63.1% were 19–25 years old; 19.4% were 26–35 years old; 8.3% were 36–45 years old; 5.1% were 46–55 years old; 0.6% were aged 56 or above.
- Marital status—15.3% were single; 67.8% were married without children;16.9% were married with children.
- Education—3.2% were high school graduates and below; 14.0% were college-educated of which, 28.3% had bachelor degrees and 54.5% had a master degree or above.
- Monthly income range (MIR)—49.4% had an MIR of less than 1 million won; 22.0% had an MIR of 1–2 million won; 20.1% had an MIR of 2–3 million won; 7.6% had an MIR of 3–4 million won; 0.9% had an MIR of 5–6 million won.
- Profession—4.4% were expert or technicians; 18.5% were businesspersons; 18.8% were service personnel; 6.4% were office staff; 45.5% were civil servants; 1.3% were students;2.2% were housewives; 1.9% were freelancers.
- Travel profile—30.9% of respondents had never travelled to China; 67.2% had travelled to China 1–10 times; 1.9% had travelled to China more than 10 times.

Of the 314 respondents, 73.9% agreed that good personal protection could effectively prevent COVID-19 infection; 66.6% agreed that China paid great attention to the health of its citizens; 65.9% agreed that China has a comprehensive medical system; 42.3% believed they could travel to China without worrying about the risk of contracting COVID-19; 51.6% agreed it was safe to travel to China. Concerning travel intent during the COVID-19 pandemic, 22.3% of respondents planned to travel abroad within a year; 43.9% planned to travel abroad within three years after COVID-19 has been eradicated; 0.03% considered travelling to China (shown as Table 1).

We also asked respondents to evaluate China’s national image. Before COVID-19, the perception of China’s national image averaged 4.847; after COVID-19, the perception of China’s national image averaged 4.716. We also conducted a post-COVID-19 statistical analysis of the type of overseas tourist destinations respondents most want to visit. We found that 15% of respondents chose natural destinations; 35% chose cultural and traditional destinations; 31% chose spiritual and religious destinations; 20% chose urban and business destinations, and; 19% chose rural and countryside destinations (shown as Table 1).

## 4. Findings

### 4.1. Validity and Reliability Analysis

We used structural equation modelling software AMOS 23.0 (IBM company, Chicago, IL, USA) to verify the research framework and the method of maximum likelihood estimation to obtain empirical results. Table 4 presents the inter-correlations between the different variables tested in the model, as well as the reliability and validity of the different measures. The results of the measurement model revealed a satisfactory model fit to the data: Chi-square/df = 1.189, *p* < 0.001, CFI (comparative fit index) = 0.979, TLI (Tucker-Lewis index) = 0.977, GFI (Goodness of Fit Index) = 0.855, NFI (non-normed fit index) = 0.880, and RMSEA (root mean square error of approximation) = 0.025. The values of TLI and CFI over 0.9 and RMSEA below 0.05 confirm a good model fit. The underlying factors were all found to have acceptable levels of reliability (PCI, α = 0.962; MM, α = 0.922; CS, α = 0.908; AT, α = 0.884; SN, α = 0.880; PAE, α = 0.835; NAE, α = 0.876; DE, α = 0.819; BI, α = 0.862; PBC, α = 0.827).

Validity and reliability were assessed by examining the composite reliability and average variance extracted (AVE) for each construct [73]. The composite reliabilities ranged from 0.816 to 0.924, meeting the threshold value for the reliability of 0.60. We utilised AVE to estimate the discriminant validity of the measurement. AVE measures the amount of change captured by its term corresponding to the amount of change caused by measurement errors. The square root of the structured AVE must be larger than each structure in the model, including the correlation with other structures, to meet the requirements of discriminant validity. The AVEs for all constructs were above the suggested value of 0.50 [74]. Table 3 shows the mean standard deviations and correlations of constructs, and it also shows AVEs and the square root of the structured AVEs.

There was acceptable discriminant validity between the two constructs, indicating that the convergent validity of the 11 constructs was acceptable. Based on the above analysis, the results indicate that all the variables in this study were reliable and valid.

Table 5 presents the means, standard deviations, and correlations of the constructs. A Varimax rotation was conducted on all items of the dependent and independent measures. The standardised factor loadings of the observed variables ranged from 0.585 to 0.904 and are shown in Table 4.

### 4.2. Structural Model

**Figure 4:**
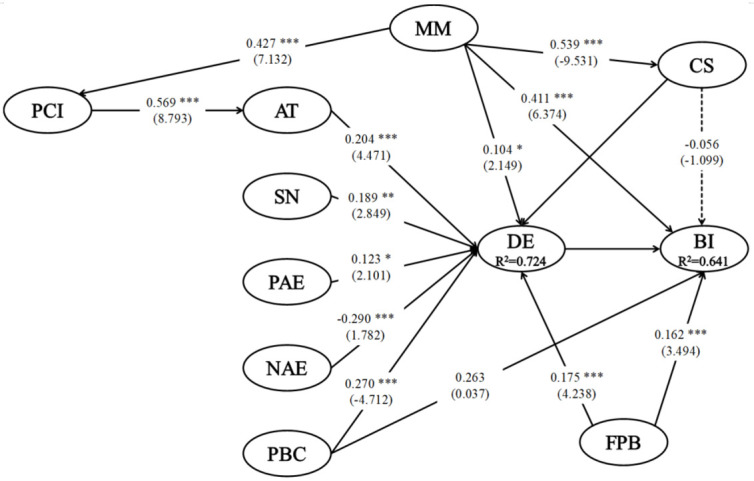
Structural model results. Note: * *p* < 0.05; ** *p* < 0.01; *** *p* < 0.001. Note 2: AT = attitude; SN = subjective norm; PAE = positive anticipated emotion; NAE = negative anticipated emotion; PBC = perceived behavioural control; PCI = perceptions of country image; MM = mass media; DE = desire; BI = behavioural intention; CS = concerns; FPB = frequency of past behaviour.

Table 6 and Figure 4 show the results of the structural model. Specifically, the results of the structural model showed an excellent fit to the data (χ2 = 1815.141, df = 1300, *p* < 0.001, χ2/df = 1.396, RMSEA = 0.036, CFI = 0.954, GFI = 0.829). Further, the results of the structural model showed that mass media (βMM→PCI = 0.427, *t* = 7.132, *p* < 0.001) had a significant positive influence on perceptions of country image, supporting H1. Mass media (βMM→CS (concerns) = −0.539, *t* = −9.531, *p* < 0.001) also had a significant negative influence on concerns, supporting H2.

Finally, mass media (βMM→BI (behavioural intention) = −0.411, *t* = 6.374, *p* < 0.001) had a significant positive influence on behavioural intention, supporting H3. Perceptions of country image (βPCI→AT (attitude) = −0.569, *t* = 8.793, *p* < 0.001) had a significant positive influence on attitude, supporting H5. Attitude (βAT→DE (desire) = −0.204, *t* = 4.471, *p* < 0.001), subjective norm (βSN→DE = −0.189, *t* = 4.471, *p* < 0.01), PBC (βPBC→DE = 0.270, *t* = −4.712, *p* < 0.05), and frequency of past behaviour (βFPB→DE = 0.175, *t* = 4.238, *p* < 0.001) had a significant positive influence on desire, supporting H6, H7, H8, H10, and H11, respectively. Negative anticipated emotion (βNAE→DE = −0.290, *t* = 1.782, *p* < 0.001) had a significant negative influence on desire, supporting H9. Desire (βDE→BI = 0.482, *t* = 5.712, *p* < 0.001) and frequency of past behaviour (βFPB→BI = 0.162, *t* = 3.494, *p* < 0.001) had a significant positive influence on behavioural intention, respectively supporting H12 and H13. However, H4 was rejected.

## 5. Discussion and Limitations

Global public health events such as SARS and H1N1 have alerted travellers to the dangers of travel-related health issues. However, the long-running nature of the pandemic is unprecedented. Humans are social beings and long-term restrictions on movement or isolation can cause psychological and mental problems. To prevent the spread of COVID-19, governments imposed restrictions on the public, in specific locations and for certain time periods. For example, in February 2020, China encouraged people to stay at home and residential communities banned entry by non-residents. Other countries also implemented lockdown and shelter-in-place policies to some extent. These restrictions on travel can be expected to lead to pent-up demand in travel that will generate a surge in travel in the post-pandemic period. However, it is also likely that the demand for travel will be tempered by the desire for travel is safe and to countries that have a proven track record in defeating SARS-CoV-2. 

In this study, we examined South Korean residents’ concerns, desires, and travel decision-making in relation to China during and after the COVID-19 pandemic. In terms of awareness about COVID-19, 74% of respondents thought that good personal protection can effectively prevent COVID-19 infection, a response that bodes well for travel. That is, as long as adequate protective measures are taken, tourists’ concerns will lessen. Concerning key survey items, 67% of respondents thought that China pays great attention to the health of its cities; 66% thought that China has a comprehensive medical system; 56% thought that the Chinese government has developed an effective medical system; 56% thoughts that the Chinese government’s control of the COVID-19 pandemic within China was effective; 42% thought that travelling to China without worrying about the risks caused by COVID-19 is possible; 53% thought that it is safe to travel to China. These results show that more than half of the respondents agree that it is safe to travel to China.

Desire is a stronger emotion than concern and supersedes it when it comes to travel. Thus, with regard to the choice of types of travel destination post-COVID-19, the results show that the desire to travel to places with natural scenery was not affected by the presence of COVID-19. The desire to travel to destinations with folk customs ranked first (35%), followed by religious sites (31%), urban business locations (20%), rural pastoral locations (19%), and natural landscapes (15%).

Hypotheses 6–13 in the original MGB were found to be tenable in this study. This indicates that the MGB can effectively predict behavioural intention. In this study, we also studied perceived country image, mass media, and concerns to improve the MGB’s capacity to explain the process of travel behavioural intention during the COVID-19 pandemic. The results verified that three additional constructs (PCI, MM, and CS) also significantly influence South Korean residents’ behavioural intention. The EMGB also helps to prevent possible misspecification, including ignoring important variables or considering unimportant variables. In other words, the three new constructs make it easier to understand the complex psychology of South Korean residents in making international tourism decisions, both during and COVID-19.

This study has some limitations. The data were collected through online platforms due to COVID-19 restrictions. Therefore, results may not be generalisable to all South Korean residents. Additionally, because of the online data-collection method, the percentage of respondents in the 19–25 years age group is 66%, much higher than other age groups in the sample. This younger group may have different behavioural intentions concerning travel overseas than older potential tourists. Future studies on this subject should test the applicability of the EMGB framework with a more evenly balanced age range and, ideally, with a much larger, randomly selected sample. With relevant modifications, the research outlined in this study could also be replicated in other countries and regions.

## 6. Conclusions

Tourism can be described as an industry that is highly sensitive to public health crisis events and one where the media plays a large role in purchasing intentions. The rapid decline in domestic and international tourist arrivals during COVID-19 supports this view. The post-COVID-19 recovery of tourism will be determined by a combination of external factors such as reopening of national borders and the effectiveness of vaccines and consumer-related factors including the desire to resume travel and concerns over health conditions in travel destinations. On the basis of MGB, this study added new constructs and focuses on the role of individual internal factors that affect tourist intention and tourist behaviour. A future study examining how these findings can be applied in other countries will be meaningful from an academic and practical perspective.

The present study has the following theoretical and practical implications. From a theoretical perspective, the prediction of international tourists’ behavioural intention towards China using the EMGB is well supported. The results of the comparison between the MGB and EMGB show that the original MGB was not an effective tool for understanding respondents’ behavioural intentions in the context of COVID-19. The EMGB is an improvement over the MGB by adding new constructs to more comprehensively analyze tourists behaviour intention. This approach to building on and modifying existing theory was described by Bagozzi (1992) as broadening and deepening of theory. From a practical perspective, while the COVID-19 pandemic continued to inhibit the resumption of international travel in early 2021, domestic travel has resumed in a number of countries including China, New Zealand and Australia.

In this study, we collected and analysed travel-related data from a sample of South Korean residents (our research subjects) to understand the influencing factors on their decision-making process concerning travel to China. Health concerns were a significant feature of future travel intentions. The results provide valuable insights into the role of public health in the resumption of travel following a public health crisis as well as the role that the media can play in forming opinions about the quality of public health services. From a marketing perspective, it is apparent that concerns about health must be identified and that customers will need to be convinced that their health concerns will be respected. In the case of future South Korean travel to China, it is apparent that Chinese firms and travel organisations must be prepared to ensure that the health concerns of international visitors are addressed. 

Based on these findings the following recommendations are made: firstly, tourism has become an important element in the lives of many people and after a long period of travel restrictions and partial isolation, people’s yearning and desire for travel may be greater than their fear and concern about the pandemic. Moreover, evidence that destination countries are able to offer high levels of protection against diseases is likely to lead to confidence that it is once again safe to travel outside of one’s own country. Secondly, the focus of this research is one of two countries that have similarities in their cultural background. In future research, there is scope to examine tourists from different cultural backgrounds as a path to building a more comprehensive tourism decision-making model and establish a multi-sample case base to add to our understanding of the impact of public health concerns on international tourism flows.

## Figures and Tables

**Table 1:** Summary of COVID-19 cases by countries (those with >10,000 cases).

Nation (1–40)	Cases	Nation (41–80)	Cases
The United States	19,573,874	Panama	231,357
India	10,208,725	Georgia	221,605
Brazil	7,484,285	Japan	217,312
Russia	3,050,248	Azerbaijan	214,711
French	2,559,686	Ecuador	209,355
Britain	2,288,345	Croatia	204,930
Turkey	2,147,578	The united Arab emirates	201,836
Italy	2,047,696	Bulgaria	197,716
Spain	1,869,610	Belarus	186,747
Germany	1,655,322	Lebanon	171,226
Colombia	1,594,497	Slovakia	167,523
Argentina	1,583,297	Dominican Republic	166,764
Mexico	1,377,217	Costa Rica	162,990
Poland	1,257,799	Armenia	157,834
Iran	1,200,465	Bolivia	154,349
Ukraine	1,025,989	Danish	153,347
Peru	1,007,657	Kazakhstan	151,727
South Africa	1,004,413	Kuwait	149,653
Netherlands	762,985	Qatar	143,062
Indonesia	713,365	Moldova	141,355
Czech republic	670,599	The Greek	135,456
Belgium	638,030	Guatemala	135,309
Romania	615,809	Palestinian	133,093
Chile	600,105	Egypt	132,541
Iraq	591,597	Tunisia	131,592
Canada	552,020	Lithuania	130598
Bangladesh	509,148	Oman	128,472
Pakistan	471,335	Ethiopia	122,864
Philippines	469,886	Myanmar	121,886
Morocco	432,079	Honduras	119,097
Swiss	428,197	Slovenia	114,806
Israel	401,470	Venezuela	112,316
Swedish	396,048	Bosnia and Herzegovina	109,691
Portugal	394,573	Malaysia	105,096
Saudi Arabia	362,220	Paraguay	104,422
Austria	351,892	Libya	98,381
Serbia	326,060	Algeria	98,249
Hungary	316,060	Kenya	95,923
Jordan	287,946	Bahrain	91,733
Nepal	258,181	Chinese mainland	86,955

Source: World Health Organization https://covid19.who.int/table (accessed date 28 December 2020, 10:00 a.m.).

**Table 2:** Excerpt of the results of the survey questionnaire (*n* = 314).

	**Response**	**Strongly Agree and Agree (*n*)**	**Neutral, Disagree, and Strongly Disagree (*n*)**	**Positive Answer** **(Percentage)**
**Item**				
Good personal protection can effectively prevent COVID-19 infection.		232	82	73.9%
China pays great attention to the health of its citizens.		209	105	66.6%
China has a comprehensive medical system.		207	107	65.9%
I can travel to China without worrying about the risks of contracting COVID-19.		133	181	42.3%
It is safe to travel to China.		162	152	51.6%
	**Response**	**Yes** **(*n*)**	**No** **(*n*)**	**Positive Answer** **Percentage**
**Question**				
Are you planning to travel abroad within a year?		70	244	22.3%
Are you planning to travel abroad within three years? After COVID-19, will you consider travelling to China?		94 9	220 224	43.9% 0.03%
Preferred overseas destination post-COVID-19 (Multiple choice) □Natural destination (15%) □Historical heritage destination (15%) □Cultural and traditional destinations (35%) □Spiritual religious destinations (31%) □Urban and business destinations (20%) □Rural countryside destination (19%) □Theme park destination (18%) □Holiday resort destination (16%)				

**Table 3:** Demographic profiles of participants (*n* = 314).

Variables	*n*	%	Variables	*n*	%
**Gender**			**Monthly income range**		
Male	106	33.8	Less than 1 million won	155	49.4
Female	208	66.2	1–2 million won	69	22.0
**Age**			2–3 million won	63	20.1
Less than 18	11	3.5	3–4 million won	24	7.6
19–25	198	63.1	5–6 million won	3	0.9
26–35	61	19.4	more than 6 million	0	0
36–45	26	8.3	**Current occupation**		
46–55	16	5.1	Expert or technician	14	4.4
More than 56	2	0.6	Businessperson	58	18.5
**Marital status**			Service	59	18.8
Single	48	15.3	Office staff	20	6.4
Married w/o children	213	67.8	Civil servant	143	45.5
Married with children	53	16.9	Student	4	1.3
**Education**			Housewife	7	2.2
High school and below	10	3.2	Freelance	9	2.9
College	44	14.0	Retired	0	0
Bachelor degree	89	28.3	Other	0	0
Master degree or above	171	54.5			
If you have traveled in China so far, you have ( ) times	0	97	30.9%		
	1–10 >10	211 6	67.2% 1.9%		

**Table 4:** Means, SD (standard deviations), and correlations of the constructs.

Constructs	Mean	SD	PCI	MM	CS	AT	SN	PAE	NAE	DE	BI	PBC
PCI	2.808	0.887	0.611 (0.782)									
MM	2.975	1.234	0.395 **	0.752 (0.867)								
CS	2.882	0.893	−0.232 **	−0.516	0.558 (0.747)							
AT	2.760	1.091	0.543 **	0.342 **	−0.139 **	0.664 (0.815)						
SN	2.651	1.168	0.362 **	0.256 **	−0.115 **	0.587 **	0.723 (0.850)					
PAE	2.824	1.091	0.306 **	0.216 **	−0.107 **	0.466 **	0.487 **	0.643 (0.802)				
NAE	2.874	1.172	−0.269 **	−0.306 **	0.137 **	−0.406 **	−0.537 **	−0.375 **	0.708 (0.841)			
DE	2.873	1.204	0.439 **	0.418 **	−0.222 **	0.610 **	0.663 **	0.529 **	−0.608 **	0.690 (0.831)		
BI	2.908	1.142	0.408 **	0.603 **	−0.356 **	0.431 **	0.479 **	0.369 **	−0.408 **	0.662 **	0.643 (0.802)	
PBC	2.901	1.067	0.291 **	0.270 **	−0.077 **	0.441 **	0.513 **	0.447 **	−0.435 **	0.599 **	0.414 **	0.629 (0.793)
**α**			0.962	0.922	0.908	0.884	0.880	0.835	0.876	0.819	0.862	0.827
**CR**			0.862	0.924	0.910	0.887	0.886	0.843	0.880	0.816	0.843	0.835

The result of the full model. CMIN = 1815.141, DF = 1300, Chi-square/df = 1.396, GFI = 0.829, RMSEA = 0.036, IFI = 0.955, CFI = 0.954. Note: **p < 0.01. AT = attitude; SN = subjective norm; PAE = positive anticipated emotion; NAE = negative anticipated emotion; PBC = perceived behavioural control; PCI = perceptions of country image; MM = mass media; DE = desire; BI = behavioural intention; CS = concerns.

**Table 5:** Confirmatory factor analysis and factor loadings.

Ten Factors and Scale Items	Standardised Loading
**Factor 1:PCI**	
Chinese politics are stable.	0.745
China is a democratic country.	0.758
China is a united country.	0.759
The Chinese economy is well developed.	0.762
China has a high level of modernisation.	0.798
Chinese technology is advanced.	0.744
China has played an important role in international affairs.	0.756
The Chinese have a high standard of living.	0.722
The Chinese have a high educational level.	0.724
The Chinese are warm and friendly.	0.758
The Chinese are polite.	0.763
The Chinese are honest.	0.769
The Chinese are diligent.	0.743
China pays great attention to environmental issues.	0.741
China has taken strict policies and measures to control environmental pollution.	0.742
China has made positive efforts to protect the environment.	0.739
China has friendly relations with South Korea.	0.722
China maintains close relations in both politics and economics with South Korea.	0.759
China and South Korea have similar cultural traditions.	0.895
**Factor 2:Attitude**	
Travelling to China is not a positive thing.	0.767
Travelling to China is not good for me.	0.780
Travelling to China is not attractive to me.	0.822
Travelling to China is not worthwhile for me.	0.885
**Factor 3:Subjective norm**	
My family does not support my travelling to China.	0.795
My friends do not support my travelling to China.	0.838
Nobody wants to travel with me to China.	0.913
**Factor 4:Perceived behavioural control**	
My budget is not enough to support my travel to China.	0.741
I do not have enough spare time to travel to China.	0.617
My health problems prevent me from travelling to China.	0.528
**Factor 5:Positive anticipated emotion**	
Travelling to China will make me happy.	0.756
Travelling to China will make me satisfied.	0.594
Travelling to China will make me thrilled.	0.578
**Factor 6:Negative anticipated emotion**	
If I could not travel to China, I would get angry.	0.749
If I could not travel to China, I would feel disappointed.	0.693
If I could not travel to China, I would be sad.	0.682
**Factor 7:Desire**	
I look forward to travelling to China soon.	0.650
I hope that I can travel to China soon.	0.729
**Factor 8:Behavioural intention**	
I am planning to travel to China soon.	0.585
I will try to travel to China soon.	0.632
I intend to travel to China soon.	0.710
**Factor 9:Mass media effect**	
The media reported on the effective controls of the Chinese government in the prevention and treatment of COVID-19.	0.746
The media reported on the safe controls of COVID-19 in China.	0.770
The media reported on the massive efforts of the Chinese government in the treatment of COVID-19 patients.	0.711
The media reported on the Chinese government’s efforts to develop a vaccine for COVID-19 prevention.	0.780
**Factor 10:Personal concerns**	
COVID-19 has made me feel isolated.	0.904
COVID-19 has made me concerned about handshakes and hugs.	0.738
COVID-19 has made me want to keep a distance from others.	0.752
COVID-19 has made me less willing to travel and participate in outdoor activities.	0.718
Travelling can be scary.	0.708
I am becoming upset.	0.737
I feel so lonely.	0.682
If I go out, I think I will be very vulnerable to COVID-19.	0.715

Note: All standardised factor loadings are significant at *p* < 0.001.

**Table 6:** The results of the structural model.

Hypothesis	Path 1	Standardised Coefficient	*t*-Value	Results
H1	MM-PCI	0.427 ***	7.132	Supported
H2	MM-CS	−0.539 ***	−9.531	Supported
H3	MM-BI	0.411 ***	6.374	Supported
*H4*	*CS-BI*	*−0.056*	*−0.110*	*Rejected*
H5	PCI-AT	0.569 ***	8.793	Supported
H6	AT-DE	0.204 ***	4.471	Supported
H7	SN-DE	0.189 **	2.849	Supported
H8	PAE-DE	0.123 *	2.101	Supported
H9	NAE-DE	−0.290 ***	1.782	Supported
H10	PBC-DE	0.270 ***	−4.712	Supported
H11	FPB-DE	0.175 ***	4.238	Supported
H12	DE-BI	0.482 ***	5.712	Supported
H13	FPB-BI	0.162 ***	3.494	Supported

Note: * *p* < 0.05; ** *p* < 0.01; *** *p* < 0.001. Italic font: the hypothesis is rejected.

## Data Availability

The study did not report any data.

## Supplementary Materials

The following are available online at https://www.mdpi.com/1660-4601/18/5/2542/s1, List S1: Lockdown Information Resources.
