# Supplementary material for: Concerns or Desires Post-Pandemic: An Extended MGB Model for Understanding South Korean Residents’ Perceptions and Intentions to Travel to China
Source: Int J Environ Res Public Health. 2021 Mar 4;18(5):2542. doi: 10.3390/ijerph18052542 (PMC7967321; doi:10.3390/ijerph18052542)
Supplement: Supplementary file 1 [file ijerph-18-02542-s001.pdf]

List S1. Lockdown Information Resources.

1. Announcement of Wuhan New Type Pneumonia Prevention and Control Headquarters (No. 1). Available online: https://www.hbtcm.edu.cn/info/1049/10179.htm (accessed on 16 November 2020).
2. Report on COVID-19 in Vinh Phuc Province, Vietnam. Available online: https://cn.qdnd.vn/cid-6149/7237/nid-567982.html (accessed on 16 November 2020).
3. Mongolian Geri. Mongolia Decided to Lift the Lockdown Two Hours ahead of Schedule. Available online: https://mp.weixin.qq.com/s?src=11&timestamp=1591155802&ver=2377&signature=H*GpfUbo-Ue6PEGuOxCRGMj6FCGN-5xHOWEdM2IHfu74-3z7rlC6YPwlYHMYniz8V9--I-WCEVHcu4ZA3xKsvR-ckTWouLGgZs4qkox70i9LRRDLi8dfcSsIZryHZq3v&new=1 (accessed on 16 November 2020).
4. Bethlehem "Closed the City": The Tacit Understanding of the High Wall behind the Chin of the Epidemic. Available online: http://www.thepaper.cn/newsDetail_forward_6453800 (accessed on 16 November 2020).
5. The First Two Confirmed Cases in Maldives: Closure of Gulido Island. Available online: https://old.dotdotnews.com/2020/03/08/breakingnews/ (accessed on 16 November 2020).
6. Measures to Prevent Novel Coronavirus Pneumonia Spread in the Eastern Region of Saudi Arabia. Available online: https://www.sohu.com/a/378561843_162522 (accessed on 16 November 2020).
7. Nearly 40 Countries or Regions Have Declared a State of Emergency and Have Successively Closed Their Countries and Cities, and the Impact on Cross-Border Logistics Is Beginning to Appear. Available online: http://www.port.org.cn/info/2020/205030.htm (accessed on 16 November 2020).
8. Austria Expands the Scope of the "Closed City", the Number of Confirmed Cases of New Coronary Pneumonia Rises to 655. Available online: http://www.xinhuanet.com/world/2020-03/15/c_1125713580.htm (accessed on 16 November 2020).
9. POLITI. New Entry Restrictions as Part of Efforts to Contain COVID-19. Available online: https://politi.dk/coronavirus-i-danmark/in-english/minstry-of-justice-12-03-2020 (accessed on 16 November 2020).
10. The Three ASEAN Countries "Lock the Country and Close the City", the Epidemic Cannot Be Ignored. Available online: https://fcg.focus.cn/zixun/a73231f92ea808f2.html (accessed on 16 November 2020).
11. More than 60 Countries around the World Have Entered a State of Emergency, and Some Countries Have Been "Closed". Available online: https://www.thepaper.cn/newsDetail_forward_6679293 (accessed on 16 November 2020).
12. Top News in Thailand. The Official of Wulinan Prefecture Ordered the Closure of the City. Available online: https://www.thaiheadlines.com/ (accessed on 16 November 2020).
13. Overseas Epidemic Tracking in the Past 24 Hours, what Places in the World Have Been Closed? Available online: https://www.shobserver.com/news/detail?id=225584 (accessed on 16 November 2020).
14. Ukraine Bans Entry of Foreigners and Suspends Regular International Passenger Transport. Available online: https://www.guancha.cn/internation/2020_03_15_541789.shtml (accessed on 16 November 2020).
15. The "Happiest Country"in the World Has Been Swept by the Epidemic. How Did Finland Fight the Epidemic? Available online: https://k.sina.com.cn/article_6013778657_16672fae100100mx9d.html?from=news (accessed on 16 November 2020).
16. Affected by the Epidemic, Almaty, the Capital of Kazakhstan, Will Be "Closed". Available online: https://www.ciwei.com/news/detail-24073.html (accessed on 16 November 2020).
17. Macau Bans Entry of Non-Local Residents from the 18th, Except for Residents of the Mainland, Hong Kong and Taiwan, and Foreign Employees. Available online: http://www.xinhuanet.com/gangao/2020-03/17/c_1125725163.htm (accessed on 16 November 2020).
18. Reference News: Argentine President Announced Mandatory Quarantine from the 20th to the 31st. Available online: https://www.sohu.com/a/381548166_114911 (accessed on 16 November 2020).
